# Compliance with water advisories after water outages in Norway

**DOI:** 10.1186/s12889-019-7504-8

**Published:** 2019-08-29

**Authors:** Susanne Hyllestad, Lamprini Veneti, Annechen Bahr Bugge, Thea Grav Rosenberg, Karin Nygård, Preben Aavitsland

**Affiliations:** 1 Department of Zoonotic, Food- and Waterborne Infections, Norwegian Institute of Public Health, Oslo, Norway; 2 Consumption Research Norway, Oslo Metropolitan University, Oslo, Norway; 3 Faculty of Medicine, Institute of Health and Society, University of Oslo, Oslo, Norway

**Keywords:** Boil water advisories, BWAs, Public compliance, Water supply interruptions, Adherence, Drinking water, Communication, Consumer trust

## Abstract

**Background:**

Water advisories, especially those concerning boiling drinking water, are widely used to reduce risks of infection from contaminants in the water supply. Since the effectiveness of boil water advisories (BWAs) depends on public compliance, monitoring the public response to such advisories is essential for protecting human health. However, assessments of public compliance with BWAs remain sparse. Thus, this study was aimed at investigating awareness and compliance among residents who had received BWAs in Baerum municipality in Norway.

**Method:**

We conducted a cross-sectional study among 2764 residents who had received water advisories by SMS in the municipality of Baerum between January and September 2017. We analysed data from two focus group discussions and an online survey sent to all residents who had received an advisory. We conducted descriptive analyses and calculated odds ratios (OR) using logistic regression to identify associations of compliance and awareness with demographic characteristics.

**Results:**

Of the 611 respondents, 67% reported that they had received a water advisory notification. Effective compliance rate with safe drinking water practices, either by storing clean drinking water or boiling tap water, after a water outage was 72% among those who remembered receiving a notification. Compliance with safe drinking water advisories was lower among men than women (OR 0.53, 95% CI 0.29–0.96), but was independent of age, education and household type. The main reason for respondents’ non-compliance with safe water practices was that they perceived the water to be safe to drink after letting it flush through the tap until it became clear.

**Conclusions:**

Awareness of advisories was suboptimal among residents who had received notifications, but compliance was high. The present study highlights the need to improve the distribution, phrasing and content of water advisory notifications to achieve greater awareness and compliance. Future studies should include hard-to-reach groups with adequate data collection approaches and examine the use of BWAs in a national context to inform future policies on BWAs.

**Electronic supplementary material:**

The online version of this article (10.1186/s12889-019-7504-8) contains supplementary material, which is available to authorized users.

## Background

Water-related diseases remain a major contributor to the global burden of disease, with 842,000 deaths annually in low- and middle-income countries [1]. In high-income countries, several outbreaks of disease associated with drinking contaminated water are reported yearly despite precautionary actions taken by water suppliers [2]. Contamination of water sources and lack of adequate water treatment are common causes, but there is increasing awareness that deficiencies in water distribution systems also represent a risk factor for (re)contamination of treated drinking water [3], not only during intermittent supply or sudden breaks [3] but also during routine maintenance operations [4]. Furthermore, measures aimed at ensuring hygienic conditions during operations to reduce the risk of gastrointestinal illness when reconnecting the water supply have been inadequate at times [5].

Boil water advisories (BWAs) are widely used to prevent the spread of illness via contaminated water. However, their effectiveness is highly dependent on public compliance [6]. A recent review of compliance with BWAs evinced 97% awareness and 76% compliance based on 11 studies [6]. The studies mainly focused on acute water incidents [7–10] or natural disasters [11–13] and rarely on planned or less acute issues with the water supply system [14]. For instance, an ageing water distribution network that is vulnerable to breaks and leakages contributes to increases in the distribution of BWAs [15].

Detection of *E. coli* in a routine monitoring scheme is the most obvious trigger of a BWA. Other situations that may trigger a BWA [16] include substantial deterioration in source water quality, major failures in treatment processes or main breaks resulting in zero or negative pressure [15]. The World Health Organization (WHO) advises water suppliers and public health authorities to develop protocols for BWAs before an emergency event occurs to avoid having to develop a response during an event, as this may complicate decision-making, compromise communication and undermine public confidence [16]. Canada, Australia and the United States [17–19] are among the few countries with a national policy on BWA use. In Canada, BWAs are categorised by cause; the detection of pathogens or indicator bacteria is often termed as an *emergency* boil water advisory, whereas water main breaks or maintenance that leads to pressure loss would be termed a *precautionary* boil water advisory [6, 14]. Evidence from Canada suggests that BWAs are more often issued due to failures in water processing and distribution than due to the detection of *E. coli* [6, 20]. The United States Environmental Agency has suggested formulating BWAs for various scenarios, such as pipe breaks [19, 21]. In Norway, the decision to issue a BWA is made by the water supplier usually in conjunction with the municipal public health authority. The Norwegian Institute of Public Health has issued general advice on the use of BWAs [22], but a comprehensive policy and national monitoring of BWA use are lacking.

In addition to concerns about BWA effectiveness, BWAs may have negative consequences, such as increased consumer anxiety and altered perceptions of drinking water quality [16, 23]. Thus, there have been calls for more monitoring and reporting of the public response to BWAs to increase understanding and improve compliance [6], particularly regarding reasons for non-compliance and perceptions of the notifications [14].

In the municipality of Baerum, Norway, the water supplier issues precautionary BWAs after planned and unplanned water outages. Between 2003 and 2016, SMS notifications were sent to residents in affected areas due to 150–200 interruptions per year in the water supply. The present study was aimed at assessing awareness, compliance, reasons for non-compliance and perceptions among residents of Baerum municipality who had received BWAs in 2017.

## Methods

We conducted a cross-sectional study among residents of the municipality of Baerum who had received water advisories by SMS between January and September 2017. We analysed the findings from two focus group discussions and data from an online survey that was administered to all residents who had received water advisories.

### Study site

The municipality of Baerum is located near Norway’s capital, Oslo, and has 124,000 residents. The municipality’s drinking water is produced in three water treatment plants – two large and one small. Although the drinking water in Baerum is considered to be of good quality, episodes of pressure drops due to breaks and maintenance occur. Municipal health authorities issue a BWA concerning water outages that last longer than 30 min, advising consumers to boil tap water for drinking and food preparation for the next 24 h. The notice reaches consumers mainly by SMS or voice message, and sometimes on the internet, and it announces any planned outages in the water supply (e.g. for maintenance) and advisories regarding the use of tap water.

**Fig. 1:**
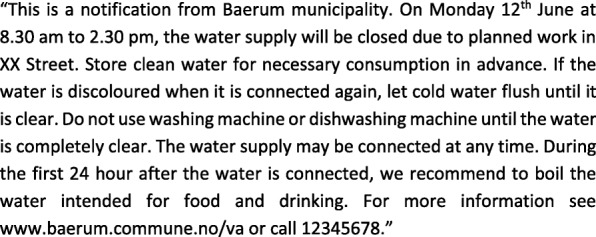
Example of a precautionary notification of a planned water outage in Baerum municipality (translated from Norwegian)

The content of a message about a water outage follows a standard format and includes the time and place of the water outage, four action points (water advice), a link to more information on the municipality’s website and a contact phone number (Fig. 1).

### Study population

The study population included individuals belonging to households connected to the public water supply in Baerum, Norway, who had been sent a BWA due to a water outage from the municipality between January and September 2017. During this period, 8091 residents of Baerum (including children) were registered as affected by 153 water outages in the municipality. Of the 153 water outages recorded, 83% were due to planned maintenance work. About 6285 notifications were sent to residents (excluding children); specifically, 5222 (83%) were sent via SMS and 1063 (17%) were sent via voice message to residents registered with landline phone numbers.

The affected population was identified by a geographic information system (GIS), and the municipality obtained contact information from the National Registry. The municipality had a list of issued notifications, which consisted of names, addresses, phone numbers and the mode of communication (SMS or voice message). From this list, we removed those who had received only voice messages to a landline. We also removed notifications sent to addresses belonging to schools, businesses and other non-individual recipients. Finally, we included persons only once even if they had received more than one notification during the period under study. After completing this process, 2764 persons remained on the list.

### Data collection and analysis of focus group discussions

From the list, residents over 70 years of age and those with children under 12 years of age were randomly invited to participate in the discussions. The group profiles were selected to represent priority audiences for the notifications. These were divided into two focus groups – one with elderly participants and one with families with children – with seven participants in each group. Both groups participated in one focus group discussion session.

A researcher moderated the discussions using a focus group discussion guide. The group discussions began by sending the participants a notification by SMS that resembled an actual notification sent from the municipality. Each focus group discussion lasted 1.5 h and was tape-recorded. After the data collection was completed, a public health/water supply researcher observed the discussions and answered questions that had been raised during the discussions.

Both of the taped discussions were transcribed into a written document. Participants’ quotations were categorised and coded in different colours according to the research questions in the study.

### Data collection and analysis of the survey

We employed the findings from the focus groups to develop an online survey about awareness of, and compliance with, BWAs. The questionnaire was developed for this study (Additional file 1 Questionnaire). The survey was sent as a link via SMS to all 2674 residents on the list. More than one person per household was invited, and up to three reminders were sent to non-responders.

We conducted descriptive analyses and calculated odds ratios (OR) using a logistic regression to identify associations of compliance and awareness with demographic characteristics. A statistical analysis was performed by Stata version 15.1 (by StataCorp).

The Data Protection Officer at the Norwegian Institute of Public Health waived the need for ethical approval according to national regulations (The Act on medical and health research of 20 June 2008) since the study did not collect personal health data and the participants to the survey remained anonymous. The need for ethical approval for the conducting the focus groups was waived to the same act. The respondents to the survey consented by filling out the questionnaire after reading the introductory text. No participants below 16 years were invited to the study. The need for written consent for the participants in the focus groups was waived according to the Act of 14 April 2000 relating to the processing of personal data (Personal Data Act) since the data collection did not contain any person sensitive data. The participants in the focus groups provided verbal consent and an email with confirmation of the verbal consent was sent to each participant who had given the consent to be a part of the study. In this email, the object of the study was repeated and the procedures for the data collection (tape recording) in the focus group was explained. The correspondence of the email with conformations of verbal consent were filed on a secure server only accessible for the responsible recruiter. The participants and their contact information provided to the study, were decoded and the file connecting the participant to their contact information were stored separately and deleted 6 months after the data collection had found place. At the beginning of the focus group discussions, the objectives of the study and means of data collection were explained again to each participant in the focus groups. The participants were assured of the anonymity and confidentiality of their responses. They were also explained that their participation were voluntary and that they could withdraw at any time.

## Results

### Findings from the focus group discussions

#### Sample characteristics of the focus groups

The two focus group discussions were held in September 2017 in the municipality of Baerum: one with seven individuals over 70 years old and one with seven families with children (below 12 years). The first focus group was composed of four women and three men ranging in age from 71 to 84 years. Four participants had higher education (university level) and three had completed high school. In the group of families with children, five were women and two were men ranging in age from 29 to 51 years. All participants in the family group had higher education (university level) and were married or cohabitants.

#### Communication, compliance and trust

Both focus groups expressed that they used smartphones actively and preferred SMS as a communication mode. The participants with children mentioned that they also received other information from the municipality at the same number, making it difficult to determine important information from the municipality. Therefore, messages about water outages were easily missed. Most participants were satisfied with the content of the messages and found them understandable with a sufficient amount of information. However, some called for more details about why the water should be boiled, but elderly women did not want more such information because they claimed that it would create ‘unnecessary fear’. Participants in the elderly group revealed uncertainties about how they should in fact ‘boil the water’.

A desire for accurate information about BWAs was expressed more clearly in the family group than in the elderly group. Few had visited the municipality’s website to obtain additional information. However, both groups appreciated that the SMS contained a link to retrieve more information from the municipality in case questions arose. As one participant expressed, ‘For the majority, knowledge gains trust… for many – that is – not for all’.

Several participants had stored sufficient amounts of clean water for drinking to last throughout the first day of a water outage. However, some believed that it was ‘not vital’ to boil tap water and did not perceive the word ‘recommendation’ as strong advice. Other participants stated that it was important to avoid becoming ‘too anxious’. In both groups, several participants had chosen not to boil their water, as they believed that letting it flush through the tap for some time was sufficient to make it safe to drink.

The participants in both groups expressed that they generally had a high degree of trust in the drinking water in Norway and, thus, had little concern related to this. They perceived the water to be ‘fresh, clean and with good taste’. However, some older participants suggested that work on the water pipes might hamper water quality. Both groups clearly expressed that the messages conveyed by the municipality did not decrease their trust; rather, the communication increased trust in the municipality regarding the water supply.

### Survey results

#### Sample characteristics

Out of the 2674 residents that were invited to complete the survey, 611 responded (response rate of 22%). Of these 611 respondents, 47% were men, 70% were above 45 year old and 85% had higher education. Regarding household type, the majority were couples with (45%) or without (37%) children in the home and 15% of the households had at least one child under the age of five (Table 1).

**Table 1:** Demographic characteristics of survey participants, municipality of Baerum, Norway, 2017 (*N* = 611)

Characteristics	Survey population # (%)
Gender	
Male	285 (47)
Female	327 (54)
Total	611 (100)
Age group	
16–35	65 (11)
36–45	120 (20)
46–65	328 (54)
> 65	98 (16)
Total	611 (100)
Highest level of education completed	
Primary school	8 (1)
High school	84 (14)
University/college (1–3 years)	171 (28)
University/college (4 years or more)	348 (57)
Total	611 (100)
Household type	
Single without children in the household	69 (11)
Single with children in the household	41 (7)
Couples without children in the household	223 (37)
Couple with children in the household	278 (45)
Total	611 (100)
Household members	
Pregnant	5 (1)
Children < 5 years old	89 (15)
Breastfeeding	15 (3)
None of the above	517 (85)
Total	NA
Number of notifications received during the previous 12 months*	
1–2 times	284 (69)
3–5 times	81 (20)
> 5 times	9 (2)
Does not know how many notifications	38 (9)
Total	412 (100.0)

*412 of 611 reported to have received a notification from the municipality

Furthermore, 412 respondents (67%) remembered receiving a BWA during the period under study (Table 1).

The majority (69%) remembered receiving an advisory one or two times during the period in question and only 2% remembered receiving more than five notifications.

#### Communication of the notifications

The majority (97%) of participants who remembered receiving a notice reported that they had received it by SMS. However, some participants had also learned about the water outage from a leaflet in their mailbox (22/412, 5.3%), from other persons in the household (11/412, 2.7%), acquaintances/neighbours (8/412, 1.9%) or other sources (5/412, 1.2%). Only a few reported learning about the water outage on social media (1 respondent) or in the newspaper (2 respondents).

SMS was the most preferred method of communication (97%). Moreover, SMS was preferred slightly more among participants who remembered receiving a message (99%) than those who did not (93%) (Table 2).

**Table 2:** Preferred sources of water advisories for future communications, municipality of Baerum, Norway, 2017

	Preferred way to be informed in the future		
Information media	Number (%) out of all participants, *N* = 611	Number (%) out of people who remembered receiving a notice, *n* = 412	Number (%) out of people who did not remember receiving a notice, *n* = 199
Mobile (SMS)	590 (97)	406 (99)	184 (93)
Leaflet in the mailbox	28 (5)	20 (5)	8 (4)
Municipality website (www.)	30 (5)	21 (5)	9 (5)
Social Media (e.g., Facebook, Twitter)	14 (2)	10 (2)	4 (2)
Email	123 (20)	78 (19)	45 (23)
Letter	6 (1)	4 (1)	2 (1)
Digital mailbox	10 (2)	6 (2)	4 (2)

Note: more than one option could be selected

#### Awareness and compliance with water advice in the notification

The notification contained four pieces of advice of which awareness and compliance were assessed (Table 3). Of those who remembered receiving a notification (412/611), approximately 66% were aware of the advice to store water in advance, 51% to let the water flush until it was clear, 43% to not use the washing machine until the water was clear and 65% to boil the water before consuming it (for cooking and drinking). Compliance was 82% for the advice to store water in advance, 92% to let the water flush until it was clear, 91% to not use the washing machine until the water was clear and 81% to boil the water before consuming it (for cooking and drinking). For both awareness and compliance (effective compliance), the proportion was around 50% for each piece of advice given, except for the advice to not use the washing machine (#3), for which effective compliance was 39%.

**Table 3:** Awareness and compliance rates for advice received, municipality of Baerum, Norway, 2017

Water advice in the notification	Awareness^a^ % (n)	Compliance^b^ % (n)	Effective compliance rate^c^ (Awareness x Compliance) %
#1 Store clean water for necessary consumption in advance	66% (273)	82% (224)	54% (82 × 0.66)
#2 Let cold water flush until clear if discoloured	51% (211)	92% (195)	47% (92 × 0.51)
#3 Do not use washing machine or dishwasher until the water is completely clear	43% (178)	91% (161)	39% (91 × 0.43)
#4 Boil water before use for food and drinking	65% (269)	81% (218)*	53% (81 × 0.65)

^a^:awareness is measured among participants who remembered receiving a notice (*n* = 412)

^b^:compliance rate is measured only among respondents who were aware of each advice (number provided in the first column)

^c^:effective compliance rate is the product of awareness and compliance and capture the effect of the ones being unaware of the BWA

*Note: 169 boiled water and 49 did not boil water because they used bottled water for food and drink

Compliance regarding safe drinking water – either by drinking stored clean water, boiled water or commercially bottled water – was 72% among participants who remembered receiving a notification and were aware of its message, and 49% among all participants.

#### Behaviour of response to BWA

Of those who chose to boil their water (*n* = 182), the main reason mentioned for following the advice was to avoid getting sick from drinking the water (80%), and 37% reported that they had no specific reasons, but trusted the advice. A smaller proportion (13%) reported following the advice due to a health condition in the household (small children, pregnancy, immunocompromised). Of those respondents who were aware of the advice, but did not follow it (*n* = 231), 45% reported that they did not boil the water because they had stored clean water in advance (regarded as compliance with safe drinking water). According to 28%, the water was visually clear and, therefore, they saw no need to boil it. Nine per cent considered the risk of getting ill by drinking the water to be very low, while 6% forgot to boil the water and 5% reported that they generally drank small amounts of water from the tap. Twenty per cent could not remember why they had not followed the advice. The survey allowed multiple choices for adherence and non-adherence to the advice. Consequently, some participants (16/412) reported both following the advisories and being unaware of the advice that they had followed.

Compliance with safe drinking water advisories (combined BWA or stored clean water in advance) was lower among men than women (OR 0.53, 95% CI 0.29–0.96), but was independent of age, education and household type.

#### Perception of risks of drinking water and trust in the water supplier

Most respondents reported that they generally had a high degree of trust in the municipality’s drinking water. Only 5% (31/611) reported the quality of the water as ‘bad or very bad’. The majority had a high degree of trust in the municipality, and only 2% (12/611) reported having low or very low trust. Four per cent (24/611) expressed concern about getting ill from drinking the water, whilst the majority reported that this was something about which they had little or very little concern. The survey allowed multiple choices for adherence and non-adherence to advice. All respondents reported on trust, although some reported not remembering receiving a notification. Almost half (48%, 293/611) reported that the communication led to increased trust in the municipality’s water supply services, and 31% (189/611) reported a small increase in trust. Seventeen per cent (104/611) reported that it did not change their perception of the municipality, and only 1% (6/611) reported that the communication decreased their trust.

## Discussion

In the present study, effective compliance with safe drinking water practices by either drinking clean water stored in advance or boiling tap water was 72% among participants who reported receiving a notification from the municipality. Since 412 of the 611 participants reported receiving a notification, the notification reached 67% of the study population. When factoring in the coverage of communication, the effective compliance rate for all survey respondents becomes 49%. Given awareness, the main reason for non-compliance was the perception that the water was safe to drink after flushing it until it was clear. The notification did not hinder the respondents’ long-term perceptions of drinking water quality but increased their trust in the municipality’s water supply services.

### Awareness and compliance with BWAs

There was an awareness rate of 65% for BWAs relating to water outages from the municipality, which is lower than reported in a meta-analysis on BWAs, where awareness was calculated to a mean of 85% and median of 97% [6]. It is less likely for routine maintenance operations on the water supply distribution network – the reason for 83% of the notifications in our study – to reach the press and contribute to public awareness as compared to what would occur in severe water incidents [8, 24]. However, awareness of the advice to store clean water in advance or boil tap water was higher than BWA alone (85%).

Eighty-one per cent compliance with BWAs was reported among participants who were aware of the notification, and effective compliance of 53% was reported when awareness was factored in. Compliance is higher than in a meta-analysis (reported mean of 68% and median of 76%), but effective compliance is lower (mean of 66% and median of 68%). We found similar results for storing water for necessary consumption in advance. Compliance with the recommendation to store clean water for drinking adds to the number of respondents who drink safe water during a water outage (here 72%); however, this is only possible in situations where there is a planned water supply interruption. In an emergency, consumers would need alternatives, such as delivered water or bottled water, and could not rely on advice to store clean water in advance.

### Communication coverage

SMS was the main notification method and the most preferred method for future communication. Coverage with this communication mode was 67%, implying that 1/3 of the study population was not reached, which affects effective compliance if factored in. Findings from the focus groups also indicate that BWA messages could easily be missed among other information from the municipality. Furthermore, it is likely that participants did not recall receiving a notice 12 months prior to the survey, even though they had, in fact, received an SMS from the municipality. In addition, technical errors in sending out the notifications or with residents’ mobile phones may be a factor. An SMS may not reach persons not acquainted or comfortable with newer technology, non-Norwegian readers or travellers not registered with a permanent address in the municipality [16]. These groups rely more on personal networks to become aware of public health messages [24]. Effects of tiredness to repeated notifications (‘message fatigue’) [16, 25] seem less relevant for our study, as the recommendation is restricted to 1 day after the reconnection of the water supply.

### Behavioural change and perception of risks

Many focus group participants perceived that ‘recommendations’ are not strong advice and leave the evaluation of risk to the individual. Furthermore, survey respondents believed that it was sufficient to let the water either run until it was clear or to allow a short time for it to be safe to drink. Even though messages issued by an authority may seem very specific and precise, recipients may not perceive the risks in the same way that experts do [26]. Thus, we suggest that a better description of the risk is needed to enable the public to make informed choices for themselves [26]. Similarly, the message to ‘boil the water’ may not be specific enough [26]. More information on health risks may have a positive effect on behavioural changes and increase compliance at the household level [27].

### Effect on trust in the water supplier

BWAs pose some dilemmas for decision-makers: exposing the public to too many precautionary BWAs could make the public lose trust in the water supplier, diminish the BWA’s credibility (‘cry wolf’ scenario) or other negative consequences, such as increasing consumer anxiety and altering perceptions of water quality [6]. These findings contrast with our findings indicating that communication served as a trust-building measure. We believe that prompt and accurate information is a mitigating measure [28]. Consumers interpret extensive communication from the water supply agency as a form of control [28], which corresponds with the findings of our study.

### Strengths and weaknesses of the study

One strength of this study is the combined data collection methods of focus group discussions and a survey. The combination of qualitative and quantitative data provides different insights to the same problem (triangulation) and enhances the validity of the study [29, 30]. The focus group discussions provided valuable insights regarding the questions, language and expressions that are relevant to a target audience [31]. The findings were triangulated by researchers with different fields of expertise in the application of qualitative methods and water supply [30].

The rather low response rate (22%) may be of concern. In terms of generalisability, one might question the extent to which our results are valid for the general population that has received and SMS in Baerum municipality. Low response rates are becoming an increasing challenge in conducting surveys, yet it has been argued that the response rate of a survey may not be as strongly associated with the quality or representativeness of the survey as generally believed [32]. The low response rate in our study may have affected the results. Participants were recruited on a voluntary basis and may not represent the general population of the municipality who have received a BWA notification (selection bias). Furthermore, they may have had a greater interest in the study topic, thus affecting the results in a positive direction [33]. Recall bias may also be relevant, particularly in the survey, due to a tendency to overestimate one’s own positive behaviour. Another weakness is that the findings may not be representative of some groups, such as older individuals without mobility, non-Norwegian speakers, illiterate individuals and those without smartphones. However, this is not a result of sampling representativeness but is, instead, related to the design of the study [34]. Data collection in the form of personal interviews in participants’ homes could have contributed to filling this gap.

In our study, we used qualitative approaches to examine a particular group or phenomenon of interest – namely, the uptake of the communication of BWAs based on one municipality’s practice. Therefore, the generalisability of the findings may be claimed to not be an expected attribute of the study per se [30]. Although the results of a quantitative study may not be directly generalisable, we believe that the results of our study are of general interest to a larger audience – in particular, water supply agencies. The study illustrates that BWAs, when issued in an informative and transparent way, may increase public trust. This is in contrast to other reported effects of BWAs [16]. The findings of the study also provide a better understanding of adherence to BWAs – an area where data are sparse [6] – and may make a valuable contribution to increasing interest in knowledge synthesis in qualitative research [30].

### Implications of the study and future research

A suboptimal awareness of BWAs affects effective compliance and implies that there exists a health risk due to possible infection. Therefore, efforts to improve awareness of BWAs are needed.

In Norway, no national policy on the use of BWAs as a precautionary measure to avoid infection risk from the water supply exists, except when *E. coli* is detected in water samples. As the health effects of an ageing water infrastructure are of national concern, there is a need to consider adopting an overarching policy regarding the use of BWAs in situations where drinking water contamination is suspected. If the water suppliers are reticent to use precautionary BWAs due to concerns about decreasing the population’s trust in the water supply, this study provides a reassuring response to such concerns. As we do not have knowledge of the practices of BWAs elsewhere in Norway, the findings of this study may not be relevant to other water suppliers. An assessment of the use of BWAs among water suppliers in Norway would make a valuable contribution when considering a possible national policy on the use of BWAs. Included in such an assessment would be the practice of issuing BWAs; reasons for not considering the use of BWAs; and the wording, content and communication methods for the notifications.

## Conclusions

In our study, awareness was suboptimal among residents who had received water advisories, but compliance with the advice in notifications of the advisories was high. The study highlights the importance of the distribution, phrasing and content of water advisory notices to achieve greater awareness and compliance. The public positively perceives information on interruptions in the water supply and precautionary recommendations to boil tap water, and such information aids in fostering greater trust in water supply authorities. Future studies should include hard-to-reach groups with adequate data collection approaches and examine the use of BWAs in a national context to inform future policies on BWAs.

## Additional file

Additional file 1: Questionnaire (DOCX 26 kb)

## Data Availability

Data from this study may be made available upon request to the corresponding author. As the original participant consent process did not explicitly state that raw data might be provided to others outside of the original study team, access to raw data will only be considered after an institutional ethics review by the requesting researcher/research team. The raw data exist in Norwegian only. The questionnaire developed for the survey in the study is found in a translated version under Additional files.

## Abbreviations

BWAs: Boil water advisories
OR: Odds ratio
SMS: Short message service
