# Supplementary material for: Compliance with water advisories after water outages in Norway
Source: BMC Public Health. 2019 Aug 29;19:1188. doi: 10.1186/s12889-019-7504-8 (PMC6716804; doi:10.1186/s12889-019-7504-8)
Supplement: Supplementary file 1 — Questionnaire (DOCX 26 kb) [file 12889_2019_7504_MOESM1_ESM.docx]

Questionnaire – Compliance with water advisories after water outages in Baerum municipality, Norway

This study is about the drinking water in the municipality of Baerum. The municipality of Baerum, together with the Norwegian Public Health Institute and Oslo Metropolitan University, will investigate in how the notifications and water advice on drinking water is perceived by the inhabitants.

**[Q1] Have you been notified of problems with drinking water from the municipality of Baerum in the past year?**

Alternative answers:

[r1] Yes

[r2] No

[r3] Don't remember / don't know

**[Q2] How were you notified?**

Alternative answers:

[r1] Mobile (sms)

[r2] Municipal web site (www.)

[r3] Social media (Facebook, Twitter)

[r4] News Paper

[r5] Note in a public location

[r6] Voice message via phone

[r7] Leaflet on the mailbox

[r8] Via other in the same household

[r9] Via other aquentances/relatives

[r10] Other

[r11] Don't remember / don't know

**[Q3] How many times have you or anyone in your household been notified about drinking water problems from the municipality of Baerum in the past year?**

Alternative answers:

[r1] 1-2 times

[r2] 3-5 times

[r3] More than 5 times

[r4] Don't remember / don't know

**[Q4] What advice did the municipality give you?**

Alternative answers:

[r1] Store clean water for necessary consumption in advance

[r2] Let cold water flush until clear if discoloured

[r3] Do not use washing machine or dishwasher until the water is completely clear

[r4] Boil water before use for food and drinking

[r5] Don't remember / don't know

**[Q5] Which of the following advice did you follow?**

Alternative answers:

[r1] Stored clean water for necessary consumption in advance

[r2] Did let cold water flush until clear

[r3] Did not use washing machine or dishwasher until the water is completely clear

[r4] Boiled water before use for food and drinking

[r5] Don't remember / don't know

**[Q6] For what use did you choose to boil the water?**

Alternative answers:

[r1] Drinking water

[r2] Food preparation

[r3] Brushing of teeth

[r4] Making ice cubes

[r5] Mixing juice drinks

[r6] Washing fruits/vegetables

[r7] Other

[r8] Don't remember / don't know

[Q7] **Why did you choose to follow the recommendation to boil the water?**

Alternative answers

[r1] To avoid getting sick / ill (diarrhea, nausea etc.)

[r2] Due to my or other health conditions in the household (young children, pregnant, immune defenses)

[r3] No special reason, but relied on the recommendation

[r4] Don't remember / don't know

**[Q8] Why didn't you choose to follow the recommendation to boil the water?**

Alternative answers:

[r1] I forgot

[r2] The water was clear and ok

[r3] Had enough stored clean water, did not need to boil

[r4] Thought there was little or no risk of getting sick

[r5] Do not drink, or generally little, tap water

[r6] Don't remember / don't know

**[Q9] How easy and understandable do you think the notification from Baerum municipality was?**

Alternative answers:

[r1] Very understandable

[r2] Quite understandable

[r3] Neither understandable nor difficult

[r4] Quite little understandable

[r5] Very little understandable

[r6] Don't remember / don't know

**[Q10] When you received the notice, did you open the link to the Baerum municipality's website for more information?**

Alternative answers:

[r1] Yes

[r2] No

[r3] Don't remember / don't know

**[Q11] Did Baerum municipality's website provide sufficient information?**

Alternative answers:

[r1] Yes

[r2] No

[r3] Don't remember / don't know

**[Q12] In what way would you prefer to receive such notifications from the municipality of Bærum?**

Alternative answers:

[r1] Sms

[r2] E-mail

[r3] Letter

[r4] Digital mailbox

[r5] Leaflet in the mailbox

[r6] Social media

[r7] Municipal website

**[Q13] How high confidence do you have in the municipality's water supply?**

Alternative answers:

[r1] Very high

[r2] Quite high

[r3] Neither high nor low

[r4] Quite low

[r5] Very high

[r6] Don’t know

**[Q14] How do the notifications affect your trust in water supply?**

Alternative answers:

[r1] Much more confidence

[r2] A little more confidence

[r3] Neither more or less trust

[r4] A little less confidence

[r5] Much less confidence

[r6] Don’t know

**[Q15] How would you rate the water quality in Baerum municipality in general?**

Alternative answers:

[r1] Very good

[r2] Quite good

[r3] Neither good or bad

[r4] Quite bad

[r5] Very bad

[r6] Don’t know

[Q16] **To what extent do you worry about getting ill from the drinking water you have at home?**

Alternative answers:

[r1] Very little worried

[r2] Quite a little worried

[r3] Neither much or little worried

[r4] Quite much worried

[r5] Very much worried

[r6] Don’t know

**[Q17] How does this affect your use of water?**

Alternative answers:

[r1] Drink less water

[r2] Drink more bottled water

[r3] Boil the water often

[r4] Drink usually other things than water (juice, milk, soda etc.)

[r5] Do not drink tap water anyway

[r6] No change

[r7] Don't remember / don't know

**[Q18] Do you suspect that you, or anyone in the household, has become uncomfortable or sick from the water you have been drinking at home during the past 12 months?**

Alternative answers:

[r1] Yes

[r2] No

[r3] Don't remember / don't know

**And then some background variables for statistical use:**

**[B1] Sex**

Alternative answers:

[r1] Male

[r2] Female

**[B2] Alder**

(insert number)

**[B3] Is there anyone in the household who is pregnant, breastfeeding or is there a child under 5 years in the household?**

Alternative answers:

[r1] Pregnant

[r2] Breastfeed

[r3] Child under 5 years

[r4] No, none of these

**[B4] What is your highest completed education?**

Alternative answers:

[r1] Elementary school

[r2] High school

[r3] University / college (1-3 years)

[r4] University / college (4 years or more)

**[B5] What type of household do you live in?**

Alternative answers:

[r1] Single without children

[r2] Single with children living at home

[r3] Cohabitants without children

[r4] Cohabitants with children living at home
